# Infectious Mimicry Complicates Diagnosis in Hemophagocytic Syndrome Caused by Anaplastic Large-Cell Lymphoma

**DOI:** 10.1155/2012/968706

**Published:** 2012-06-17

**Authors:** Michael J. Peluso, David Chia, Whitney Sheen, Christoph Hutchinson, Lydia Barakat

**Affiliations:** ^1^School of Medicine, Yale University, New Haven, CT 06510, USA; ^2^Primary Care Center and Department of Internal Medicine, School of Medicine, Yale University, New Haven, CT 06510, USA; ^3^Department of Internal Medicine, School of Medicine, Yale University, New Haven, CT 06510, USA; ^4^Section of Infectious Diseases, School of Medicine, Yale University, New Haven, CT 06510, USA

## Abstract

Hemophagocytic syndrome (HPS) arises secondary to genetic, rheumatologic, neoplastic, and infectious causes. We discuss a patient whose presentation was consistent with systemic infection but was discovered to have HPS of unknown etiology. The presenting symptoms, as well as unremarkable malignancy and rheumatologic workups, led to the pursuit of an infectious cause, but the patient was ultimately discovered to have an occult anaplastic large-cell lymphoma (ALCL). This case demonstrates the diagnostic challenges that result from infectious mimicry in the context of HPS—first, in distinguishing noninfectious HPS from the systemic inflammation that can result from a widespread infectious process, second, in the identification of the precipitating cause of HPS. While evidence of these challenges has been suggested by the limited literature on HPS and ALCL, our case illustrates the diagnostic dilemma that arises when tissue biopsy does not quickly reveal an etiology. It is important that all physicians be aware that HPS can mimic infection and be prepared to redirect the workup when an infectious etiology for HPS cannot be identified.

## 1. Introduction

Hemophagocytic syndrome (HPS) is a rare, life-threatening condition associated with significant challenges in diagnosis and management. HPS can be more specifically described according to its association with a precipitating condition in each patient. It is alternatively known as hemophagocytic lymphohistiocytosis (HLH) when associated with a primary genetic disorder and macrophage-activation syndrome (MAS) when associated with a rheumatologic disorder. It can also occur secondary to malignancy (malignancy-associated hemophagocytic syndrome, MAHS) or infection (infection-associated hemophagocytic syndrome, IAHS). A physician must have a reasonable index of suspicion for HPS in patients with undiagnosed disease suggestive of systemic inflammation and must consider both infectious and noninfectious etiologies of the syndrome once the diagnosis of HPS has been made. 

We report the case of a young adult presenting to our institution with HPS that represented a challenge in the diagnosis and search for an underlying etiology. This case illustrates how the identification of HPS can be delayed when a primary infectious process is suggested by the patient history, and how infectious mimicry can postpone the consideration of malignancy as the primary cause once HPS has been identified. Although the condition is rare, it is important that physicians be prepared to consider the diagnosis of HPS in cases where the infectious workup is unexpectedly negative and be able to conduct a thorough investigation for the underlying etiology when the syndrome is identified.

## 2. Case Presentation

### 2.1. Patient Presentation and History

A 20-year-old male from Mexico with no significant past medical history presented with 3 months of left facial swelling and pain associated with fevers, night sweats, unintentional weight loss, myalgias, and arthralgias. 

He had been evaluated and treated multiple times by the emergency department, an inner city hospital, and an outpatient dentist with several different antibiotic regimens, narcotic pain control, oral steroids, and drainage catheter placement for a presumed dental abscess. His symptoms improved slightly with these interventions but recurred after the completion of each course of treatment. After his fifth admission at an outside hospital, he was transferred to our university hospital for evaluation of fever of unknown origin and workup of presumed sepsis. 

He denied visual changes, symptoms of upper or lower respiratory infection, chest pain or palpitations, nausea, vomiting, diarrhea, hematochezia, dysuria, exposure to animals or insects, sick contacts, recent travel, or high-risk behaviors for substance abuse or sexually transmitted infections. He had received all of his immunizations as a child in Mexico. He denied family history of any illnesses. 

### 2.2. Physical Examination

Upon transfer and admission to our institution, his vital signs were within normal limits: temperature of 98°F, heart rate of 69 beats per minute, blood pressure of 112/72, respiratory rate of 12 breaths per minute, and oxygen saturation of 92% on room air. On exam, he was noted to have nonmobile and tender left mandibular swelling. His eye exam showed red scleral injection without exudate and a grey-brown ring around the irises. Neck exam revealed posterior left cervical lymphadenopathy that was hard, fixed, and painful. The remainder of the HEENT exam was unremarkable. Lungs were clear to auscultation bilaterally, and the cardiovascular exam was within normal limits. The abdomen was soft, nontender, and nondistended. Pulses were 2+ bilaterally, and the neurological exam was nonfocal.

### 2.3. Laboratory Evaluation and Radiologic Studies

**Figure 1:**
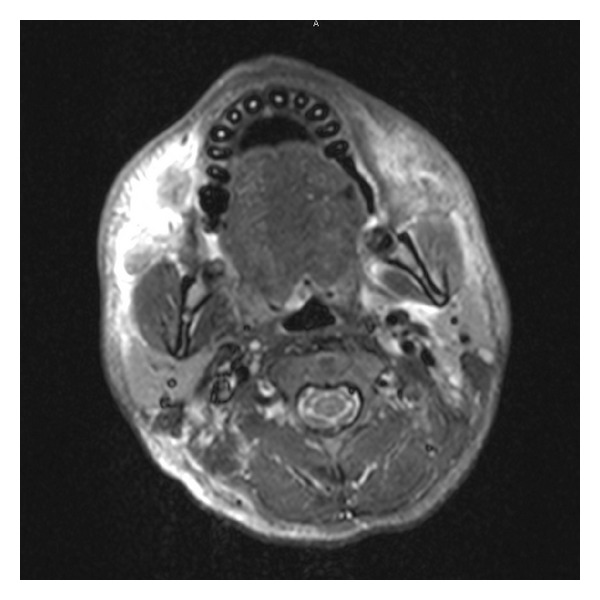
CT maxillofacial scan demonstrating extensive inflammatory changes of the left face without evidence of abscess. These changes were later determined to be nonspecific inflammatory changes resulting from ALCL-associated HPS.

Initial laboratory evaluation was notable for hyponatremia (Na 133 mmol/L, NL 135–145 mmol/L), anemia (hemoglobin 9.5 g/dL, NL 14.0–18.0 g/dL; hematocrit 30%, NL 40.0–52.0%), leukopenia (WBC 2.0 × 10^9^/L, NL 4.0–10.0 × 10^9^/L), hypertriglyceridemia (284 mg/dL, NL 30–150 mg/dL), and transaminitis (ALT 206 U/L, NL 0–34 U/L; AST 102 U/L, NL 0–34 U/L). Infectious disease evaluation on admission included multiple blood and urine cultures that were negative. A chest X-ray and computed tomography (CT) imaging of the chest showed multiple bilateral pulmonary nodules of uncertain etiology as well as a right lower lobe consolidation, paratracheal, mediastinal, and left hilar nodes, and left axillary edema. CT of the abdomen and pelvis showed bilateral pyelonephritis with enlarged spleen, and a CT maxillofacial scan demonstrated inflammatory changes of the face without evidence of abscess (Figure 1). 

### 2.4. Treatment, Clinical Course, and Outcome

The patient was started on broad-spectrum antibiotics, stress-dose steroids, and IV fluids, which resulted in hemodynamic stability but persistent fevers. Repeat infectious disease workup, including blood, urine, sputum, and fecal cultures, HIV testing, PPD, VDRL, transthoracic echocardiography, and soft tissue biopsy of the left mandible, was unremarkable. 

**Figure 2:**
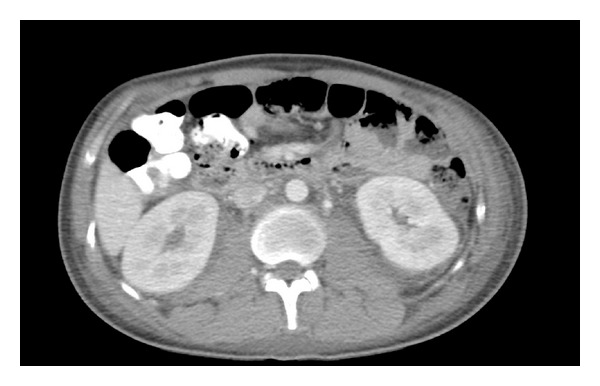
CT abdomen demonstrating extensive retroperitoneal fat stranding. These changes were later determined on autopsy to represent abdominal involvement of ALCL, which was present in mesenteric and retroperitoneal lymph nodes.

A more thorough laboratory investigation was initiated. Abnormalities included elevated LDH (1102 U/L, NL 118–232 U/L), ferritin (13,500 ng/mL, NL 18–370 ng/mL), C-reactive protein (64.6 mg/L, NL 0.1–4.9 mg/L), and erythrocyte sedimentation rate (54 mm/hr, NL 0–20 mm/hr). Repeat CT scan revealed bilateral pleural effusions, numerous pulmonary nodules, and axillary, subpectoral, and retroperitoneal fat stranding (Figure 2). A PET scan demonstrated several hypermetabolic lesions involving musculature and fat stranding throughout the body, most likely related to a systemic infectious or inflammatory process.

**Figure 3:**
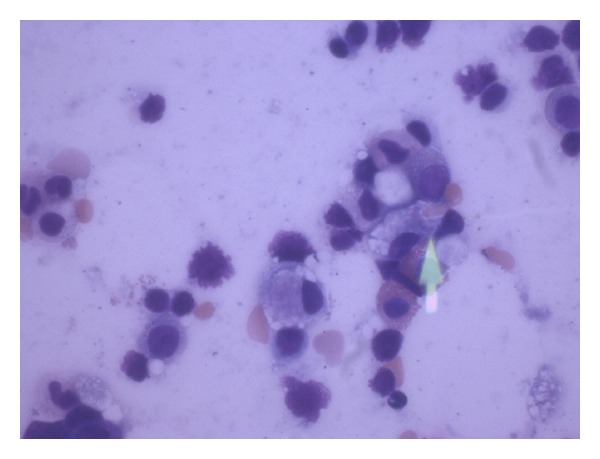
Bone marrow biopsy showing hemophagocytosis of an erythrocyte by a macrophage, pathognomonic for hemophagocytic syndrome.

A liver biopsy was suggestive of drug reaction. Lung nodule biopsy showed normal tissue in the lesions and no evidence of infection or malignancy. Left mandibular soft tissue biopsy showed necrosis and acute inflammation, but no evidence of infection or malignancy. Finally, bone marrow biopsy revealed extensive macrophage hemophagocytosis, pathognomonic for hemophagocytic syndrome (Figure 3). 

The patient was recognized to have HPS and started on IVIG and high-dose IV dexamethasone before transitioning to a slow prednisone taper and cyclosporine. An extensive search for an underlying rheumatic (ANA, ds DNA, anti-MPO, anti-PR3, RF and CCF antibodies, c-ANCA, and p-ANCA) and malignant etiology (flow cytometry, T-cell receptor rearrangement study, bone marrow, soft tissue, and lung biopsies, PET scan) was unrevealing. No evidence of malignancy was evident on any of the bone marrow or soft tissue biopsy specimens. As a result, a working diagnosis of infection-associated hemophagocytic syndrome with an unknown primary infection was pursued. However, an exhaustive infectious disease workup (including viral hepatitis, EBV, Parvovirus, HSV, CMV, HHV-6, *Coccidioides*, *Trichinella*, leishmaniasis, *Cryptococcus*, *Aspergillus*, histoplasmosis, and acid-fast *Bacilli*) was negative. 

Despite appropriate treatment for HPS, the patient deteriorated and eventually expired from a massive GI bleed. On autopsy, he was found to have occult anaplastic large-cell lymphoma (ALCL; stage IV) involving lung, stomach, lower GI tract, mesenteric lymph nodes, and spleen. 

## 3. Discussion

### 3.1. Definition, Epidemiology, and Etiology of HPS

HPS is a rare condition in which cytokine overstimulation of macrophages results in overproliferation and hemophagocytosis in bone marrow and lymphoid tissue. It is characterized by fever, splenomegaly, bicytopenia, hypertriglyceridemia or hypofibrinogenemia, marked elevation in ferritin (>500 ng/mL), high concentration of soluble interleukin-2 receptor, low NK-cell activity, and macrophage hemophagocytosis on bone marrow biopsy [1, 2]. A patient must have five conditions to meet criteria for diagnosis. 

Primary HPS [3] is associated with genetic mutations, while secondary HPS is attributed to infectious, rheumatologic, or malignant causes resulting in massive cytokine overstimulation. The disease is most commonly found in children [4, 5]. Its prognosis is poor, and mortality has been shown to approach 50% [6]. 

A comprehensive overview of case reports and case series on a wide array of infectious processes associated with HPS has previously been compiled [7] and demonstrates the breadth of infectious etiologies that must be considered by the infectious disease physician in a patient with HPS. Physicians face several challenges in conducting the evaluation for HPS in the context of a patient with a known infection. An infectious process may precipitate both primary [8, 9] and secondary forms of the condition, so it may not be immediately obvious whether the disease has a genetic or infectious primary cause. This is important because familial HPS is treated most effectively with allogenic bone marrow transplantation, while treatment of underlying infection will likely result in the resolution of IAHS [10]. While official guidelines regarding the infectious workup for HPS do not exist, we conducted a series of tests that covered many of the diseases with which HPS has been associated, including bacterial, viral, and fungal pathogens. 

### 3.2. Teaching Points of the Case

The key educational points from this case involve not infection itself, but rather infectious mimicry, the concept that a noninfectious disease can present with signs typically seen in infection, thereby encouraging the medical team to pursue a workup that does not reveal the primary disease process. Issues regarding infectious mimicry arose at both of the key diagnostic steps in this case: first, with the identification of HPS (and not sepsis) as the syndrome exhibited by the patient, second, as the etiology of the HPS, once identified, was pursued.

**Point 1: Systemic Inflammation Characterizes Both HPS and Widespread Infectious Processes**

The first diagnostic step in our case was the recognition that our patient was experiencing HPS and not only a systemic inflammatory response associated with sepsis or viral infection. Regardless of the primary cause, HPS itself is characterized by the same systemic inflammation as a widespread infectious process [8, 10]. Because both bacterial sepsis and systemic illness with a number of viral pathogens (Epstein-Barr virus, cytomegalovirus, viral hepatitis, and the human immunodeficiency virus) can display widespread inflammation, a physician might pursue an infectious diagnosis and fail to recognize the presence of HPS in the first place. In our patient, we pursued a diagnosis of sepsis and did not consider the presence of HPS until multiple infectious disease evaluations were negative, and we began to investigate noninfectious conditions.

The pursuit of a nonexistent systemic bacterial or viral infection in this case delayed the identification of HPS in the patient and thus delayed the investigation for the primary cause of HPS. In our case, the patient's presentation appeared consistent with progression of a dental abscess to severe sepsis, and this is the diagnosis that was pursued during his previous hospital admissions. It was only after multiple failed treatments for presumed infection at outside institutions that the patient was referred to our tertiary care facility, and even then, it was still thought that the patient was displaying signs consistent with systemic infection. Physicians must therefore be aware of HPS as a syndrome characterized by many of the same findings as widespread infection and consider this as a possibility when the infectious workup produces an unexpectedly negative result, especially when that result persists and the patient fails to improve with appropriate treatment for sepsis.

**Point 2: Once HPS Is Identified, Inability to Identify a Noninfectious Primary Cause Does Not Guarantee an Infectious Primary Cause**

Infectious mimicry is similarly problematic once HPS has been identified. The physician caring for a patient with HPS faces a considerable diagnostic dilemma with regard to the search for underlying processes. Our patient's disease presented with signs of sepsis and widespread systemic inflammation including cytopenia, pancreatitis, hepatitis, lung consolidations, and pleural effusions. While the pulmonary nodules were suggestive of malignancy and would be unlikely to occur in IAHS, the nondiagnostic tissue biopsies, as well as negative bone marrow, cell marker studies, and T-cell receptor rearrangement studies shifted our focus away from a malignant primary cause. We therefore defaulted once again to the pursuit of an infectious primary cause because the noninfectious investigations did not yield a malignant or rheumatologic etiology.

The fulminant course prevented the diagnosis of the underlying malignancy and resulted in the pursuit of an infectious cause that did not exist. It is important for physicians to recognize in these cases that absence of clear evidence for noninfectious etiologies of HPS should not result in an assumption of an infectious cause. Moreover, absence of infection should encourage further noninfectious workup. The investigation of a diagnostic pathway should not be suspended until an underlying etiology has been clearly identified.

### 3.3. HPS and ALCL

The etiology of HPS in this case ultimately proved to be ALCL. It has been demonstrated that ALCL is characterized by a high incidence (>50% of patients) of systemic symptoms at presentation [15]. A histological variant of ALCL (“Linda Brown tumor”) is characterized by extensive proliferation of phagocytosing histiocytes accompanying the infiltrating tumor [16]. There is evidence that ALCL secretes several proinflammatory cytokines and chemokines [17], which are likely to be responsible for inducing HPS. 

HPS has been commonly associated with T-cell lymphoma [18], and according to one review, ALCL is the dominant lymphoma in childhood leading to HPS, even though it accounts for only 10–15% of childhood non-Hodgkin's lymphoma (NHL) [19]. In adults, however, association with ALCL is rare [20]. Only a few published cases discuss patients with ALCL who meet HLH-94 and revised HLH-2004 criteria [11–14]. 

It is not uncommon for ALCL-associated HPS to be confounded by an infectious presentation (Table 1). In three cases [12, 13], initial workup suggested IAHS even though an infectious agent could not be identified. In the fourth [11], the patient was thought to have viral hepatitis. One case was identified as ALCL during the initial workup, and in this case, the patient was successfully treated [14]. It is important for clinicians to recognize that HPS, and especially ALCL-associated HPS, can present as and be mistaken for a primary infectious or infection-related process. 

In each of the previous cases, tissue biopsy revealed ALCL, and the diagnosis was made in time to treat the patient appropriately. It has been noted in one paper that ALCL-associated HPS can include the type of lung findings (mediastinal adenopathy, lung nodules) that were present in our patient [13]; however, in these cases, the tissue biopsy was diagnostic of ALCL, while in ours it was not. In our case, many of the findings were too nonspecific to facilitate a diagnosis of ALCL in time to be of benefit to the patient. The physical exam finding of painful, fixed cervical lymphadenopathy has been known to occur in ALCL but is nonspecific to this condition. Similarly, the facial swelling was characterized by nonspecific inflammatory features that did not aid in the diagnosis of ALCL, even after autopsy. The retroperitoneal fat stranding was determined to be due to abdominal involvement on autopsy but again was considered nonspecific during the imaging evaluations. While it has been shown that the liver manifestations seen in our patient can be a presenting manifestation of HPS [21, 22], these findings are also nonspecific. 

In this case, the majority of disease was in the gastrointestinal tract and surrounding tissues, a relatively rare location for ALCL [23]. Due to our patient's rapidly progressive course and unremarkable malignancy workup, we were unable to procure the necessary tissue specimens to correctly diagnose the cause of HPS as ALCL before death. 

## 4. Conclusion

The presentation of a patient with HPS represents a significant challenge for physicians conducting the diagnostic workup. Infectious mimicry suggestive of sepsis and unrevealing studies for rheumatologic and malignant etiologies may shift the differential toward infectious causes. While a thorough infectious disease workup is essential, the rheumatologic and neoplastic workup should not be suspended if an infectious cause is not immediately obvious. Identification of the underlying etiology of HPS is crucial in order to initiate timely and appropriate treatment and to avoid the fatal outcome of the disease. It appears that HPS secondary to ALCL tends to present with signs that may be mistaken for evidence of infection. It is especially important for a physician to consider this in challenging cases in which the infectious workup is unrevealing.

## Figures and Tables

**Table 1:** Comparison of the present case with previously published cases of ALCL-associated hemophagocytic syndrome.

	Present case	Krenova et al. [11]	Sovinz et al. [12]	Sevilla et al. [13]		Cho et al. [14]
Age, gender	20 years, M	17 years, F	15 years, M	16 years, F	5 years, F	25 years, M
History and physical exam	FUO, facial swelling, LAD, and uveitis	FUO, LAD, and splenomegaly	FUO, neck swelling, and splenomegaly	FUO, splenomegaly	FUO, abdominal pain, and headache	FUO, myalgia, weakness, jaundice, LAD, and splenomegaly

Labs						
WBC (cells/L)	2.0 × 10^9^	—	5.0 × 10^9^	1.4 × 10^9^	5.2 × 10^9^	1.6 × 10^9^
ANC (cells/L)	0.7 × 10^9^	0.4 × 10^9^	—	—	—	—
Hemoglobin (g/dL)	9.5	4.4	10.1	7	8.9	8.8
Platelets (cells/L)	145 × 10^3^	46 × 10^3^	167 × 10^3^	84 × 10^3^	213 × 10^3^	88 × 10^9^
Triglycerides (mg/dL)	284	316	218	—	—	113
Fibrinogen (mg/dL)	246	70	210	320	91	150
Ferritin (ng/mL)	13500	20 (g/L)	9270	5166	—	2240
Imaging studies	Soft tissue swelling, splenomegaly, fat stranding, and lung nodules	—	Cervical LAD	Mediastinal LAD, splenomegaly, and lung nodules	Splenomegaly, mediastinal and hilar LAD	Cervical, mediastinal, and abdominal LAD, hepatomegaly, and splenomegaly
Initial diagnosis	IAHS	Hepatitis	IAHS	IAHS	IAHS	ALCL

Biopsy						
Lymph node	—	ALCL	HPS; ALCL	ALCL	ALCL	ALCL
Soft tissue	Necrosis, HPS	—	—	—	—	
Bone marrow	HPS	HPS; ALCL	HPS	HPS	HPS	HPS
Lung nodule	Nonspecific	—	—	ALCL	—	—
Final diagnosis	ALCL	ALCL	ALCL	ALCL	ALCL	ALCL

Abbreviations: M: male; F: female; FUO: fever of unknown origin; LAD: lymphadenopathy; WBC: white blood cells; ANC: absolute neutrophil count; ALCL: anaplastic large cell lymphoma; HPS: hemophagocytic syndrome; IAHS: infection-associated hemophagocytic syndrome.
